# *Macadamia integrifolia* Leaf Photosynthesis and Carbohydrate Status Following Whole-Plant Flooding

**DOI:** 10.3390/plants15121779

**Published:** 2026-06-09

**Authors:** Suzy Y. Rogiers, Dennis H. Greer, Jean T. Page, Jay M. Anderson, Jeremy D. Bright, Kevin P. Quinlan

**Affiliations:** 1 Department of Primary Industries and Regional Development, Wollongbar Primary Industries Institute, Wollongbar, NSW 2477, Australia; jeremy.bright@dpird.nsw.gov.au (J.D.B.); kevin.quinlan@dpird.nsw.gov.au (K.P.Q.); 2 Faculty of Science and Engineering, Southern Cross University, Lismore, NSW 2480, Australia; jean.page@scu.edu.au (J.T.P.); jay.anderson@scu.edu.au (J.M.A.); 3 School of Agricultural, Environmental and Veterinary Sciences, Charles Sturt University, Wagga Wagga, NSW 2678, Australia; dennisgreer84@gmail.com

**Keywords:** flooding, macadamia, photosynthetic recovery, inundation, nuts, climate change

## Abstract

Extreme flooding has emerged as a major climate risk for low-lying Australian macadamia (*Macadamia* spp.) orchards, yet the physiological mechanisms underlying tree decline remain poorly understood. We investigated whole-plant responses to complete submergence in young, grafted macadamia trees by subjecting plants to one- and two-week floods, as well as repeated flooding. Following emergence from the flood water, photosynthetic rate (*A*) and stomatal conductance (*g_s_*) declined progressively with increased flood duration and repeated exposure. Grafted plants of G on H2 maintained a more resilient photosynthetic apparatus post-flood than G grafted on Beaumont, as reflected by a smaller decline in maximum assimilation rates as well as biochemical capacities for ribulose 1,5 bisphosphate (RuBP) carboxylation (*V_cmax_*), and RuBP regeneration (*J_max_*). Despite these differences in leaf-level function, prolonged and repeated flooding triggered a cascade of post-flood stress symptoms in both rootstocks, including progressive canopy dieback, sharp reductions in root biomass, depletion of total non-structural carbohydrates, and ultimately scion mortality. Collectively, these findings indicate that plants only partially tolerated one week of complete submergence, whereas longer or repeated flooding severely compromised carbon balance and plant survival in both rootstocks.

## 1. Introduction

Photosynthesis is commonly suppressed during and after flooding, constraining a plant’s energy supply [1]. When the entire plant is submerged, both roots and shoots are deprived of oxygen and light. Submerged leaves may accumulate ethylene and other stress hormones [2,3], triggering stress responses such as leaf abscission or chlorosis [4,5]. Photosynthetic machinery may be damaged [6], especially if the plant is submerged for long periods, leading to irreversible declines in photosynthesis [7]. Below ground, nutrient uptake may be inhibited [8,9] and roots may become inactive or even die as a result of hypoxia [10,11,12]. During submergence, plants may rely on stored carbohydrates because photosynthesis is suppressed [13]. However, carbohydrates may not be mobilised because oxygen is required to convert starch to transportable sucrose and, therefore, the entire plant may suffer from low energy supplies [14].

Once the water recedes, flood-sensitive plants do not instantly recover; they go through a second wave of stress that still strongly affects photosynthesis [15]. Water-damaged leaves often have reduced chlorophyll content, leading to lower light capture and weaker photosynthesis. Moreover, key proteins in the photosystems can be degraded [6]. Rebuilding these components may be slow, meaning photosynthetic efficiency remains lower than normal for weeks or even months, requiring new leaves to be developed. Stomata may not reopen properly after flooding, either staying closed or behaving erratically [16,17]. This limits CO_2_ uptake even after the water is gone, which continues to restrict photosynthesis. After submergence, when plants are suddenly exposed to high oxygen and light again, they often produce reactive oxygen species (ROS) [18,19], which are highly reactive molecules that can damage chloroplasts, membranes, and enzymes critical for photosynthesis. In some cases, plants will prioritise survival by shedding older, damaged leaves [3,20]. This reduces the total photosynthetic area, even if new leaves eventually grow back.

Flood and waterlogging tolerance is highly species dependent [13]. Macadamia trees (Proteaceae), including *Macadamia integrifolia* and *Macadamia tetraphylla*, are native to the subtropical rainforests of eastern Australia, particularly in southern Queensland and northern New South Wales [21]. In these regions, macadamia evolved under warm, humid conditions with relatively high annual rainfall, often exceeding 1000 to 2000 mm per year, distributed fairly evenly throughout seasons, with peaks during the summer months [22]. In modern agricultural settings, macadamia is sometimes planted in flood-prone areas, increasing the risk of waterlogging and plant submersion after major rainfall or extreme weather. In the 2022 growing season, unprecedented autumn rainfall caused flooding in many new orchards planted in the coastal floodplain of northern NSW. In some instances, young macadamia trees were fully submerged for over a week. Many plants did not recover and died over the following months.

Given the critical role of carbohydrates in plant survival and recovery, one of the key parameters we focused on was leaf photosynthesis following canopy submergence. The study examined how flooding duration affects CO_2_ uptake, stomatal function, and overall photosynthetic efficiency, factors that directly determine a plant’s capacity to recover from prolonged submergence. Plant carbohydrates were also assessed before and after complete submergence. The research hypothesised that longer and repeated flooding would cause incomplete recovery of photosynthetic capacity through stomatal limitation, biochemical impairment, and depletion of carbohydrate reserves. To test this, plants with G scions grafted onto H2 or Beaumont rootstocks were subjected to either one- or two-weeks of complete submergence, or two sequential floods, followed by a recovery period.

## 2. Results

### 2.1. Leaf Dry Weight and Scion Vitality at the Time of Flood Removal

Prior to the start of the experiment, G scions on H2 rootstocks (G/H2) were similar in size to G scions on Beaumont rootstocks (G/BMT) with a plant dry weight of 134.3 ± 7.6 and 123.1 ± 9.0 g, respectively (*p* = 0.350). There was no significant difference in leaf dry weight (48.3 ± 2.8 and 46.0 ± 3.6 g, *p* = 0.608), branch dry weight (13.7 ± 1.2 and 11.8 ± 1.4 g, *p* = 0.271), trunk dry weight (38.7 ± 1.7 and 35.0 ± 2.4 g, *p* = 0.210), or root dry weight (33.4 ± 2.9 and 30.4 ± 2.7 g, *p* = 0.445).

**Figure 1:**
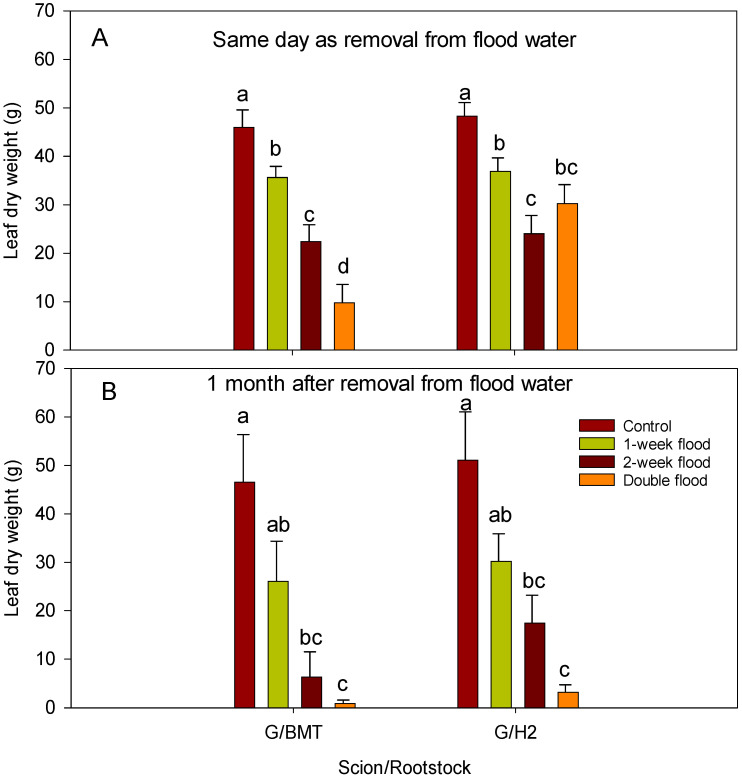
Total plant leaf dry weight of G scions on Beaumont rootstock (G/BMT) and G scions on H2 rootstocks (G/H2) in response to a 1-week flood, a 2-week flood, or a double flood treatment. The assessment was carried out within 24 h of plant removal from the water (**A**) and one month later (**B**). Bars indicate ± standard error of the means (*n* = 5). Flood treatment (*p* < 0.001) was significant but rootstock was not (*p* = 0.110). The interactive effect of treatment x rootstock was not significant (*p* = 0.183). Groups that share a letter are not significantly different, while groups with completely different letters are significantly different.

**Figure 2:**
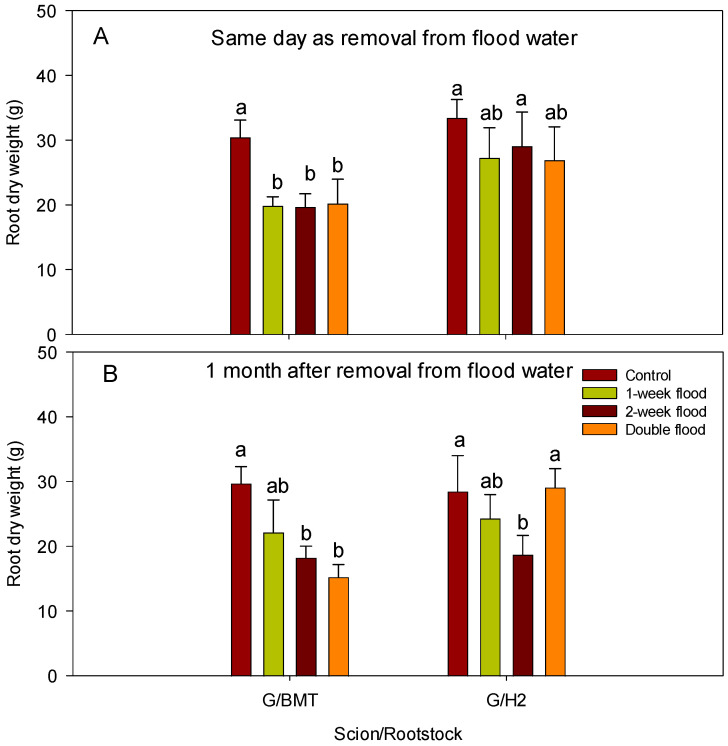
Root dry weight of G/BMT and G/H2 in response to a 1-week flood, 2-week flood, or double flood treatment. The assessment was carried out within 24 h of plant removal from the water (**A**) and one month later (**B**). Bars indicate ± standard error of the means (*n* = 5). Flood treatment (*p* < 0.05) and rootstock (*p* < 0.05) were significant. The interactive effect of treatment x rootstock was not significant (*p* = 0.830). Groups that share a letter are not significantly different, while groups with completely different letters are significantly different.

Total plant leaf dry weight decreased in response to the submersion treatments (Figure 1). Leaf dry weight was lower immediately after the removal from the 1-week (23–24% relative to the control, *p* < 0.05), 2-week (50–51% relative to the control, *p* < 0.001) and double flood treatment (37–79% relative to the control, *p* < 0.001), and this declined even further one month after the double flood treatment (93–98% relative to the control, *p* < 0.001). There was no rootstock effect on leaf loss (*p* = 0.110). There was, however, a rootstock effect on root dry weight (*p* < 0.05; Figure 2). Immediately after removing the plants from the water, root dry weight of G/BMT had declined by 34–35% in the 1-week, 2-week, and double flood treatments (*p* < 0.05). One month later, root dry weight of the 1-week treatment was not different from the control; however, it was 39% less in the 2-week and 49% less in the double flood treatment. In contrast, G/H2 did not have an immediate decline in root weight during the floods, but in the following month it was 44% less in the 2-week treatment relative to the control (*p* < 0.05).

### 2.2. Stomatal Conductance

**Figure 3:**
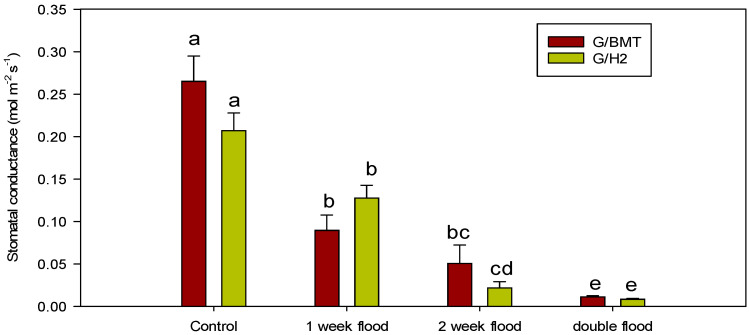
G/BMT and G/H2 leaf stomatal conductance (*g_s_*) of control plants, and of plants that had been exposed to a 1-week flood, a 2-week flood, or a double flood treatment. The assessments were conducted 1 to 2 weeks after removing the plants from the water, then irrigating them to field capacity. Bars indicate ± standard error of the means (*n* = 4). The main effect of flood treatment (*p* < 0.001) was significant. Groups that share a letter are not significantly different, while groups with completely different letters are significantly different.

Stomatal function did not recover once plants were removed from the water. Leaf stomatal conductance (*g_s_*) was lowest for those plants that had the longest duration of submersion (*p* < 0.001; Figure 3), with no rootstock differences (*p* = 0.865). The 1-week flood resulted in 38–66% lower *g_s_* than the control, and 81–90% lower values because of the 2-week flood treatment. The double flood treatment resulted in almost complete stomatal closure, at 95%, in both rootstocks.

### 2.3. Effect of Internal CO_2_ on Assimilation

**Figure 4:**
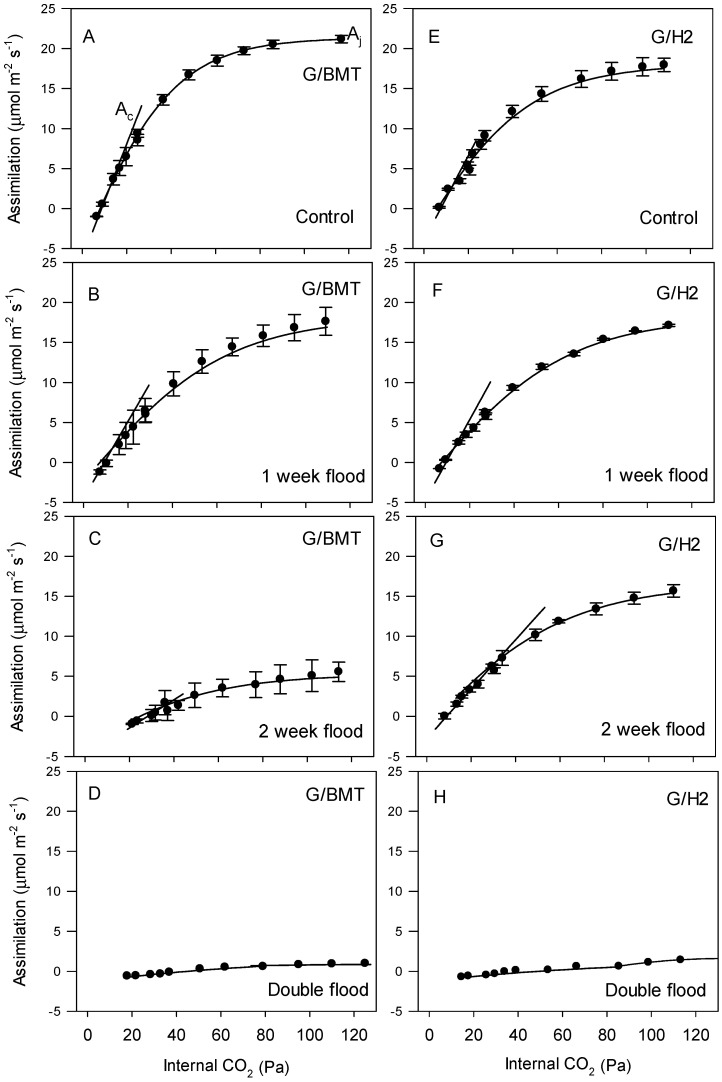
Assimilation in relation to the internal CO_2_ concentration of G/BMT and G/H2. In each case the lines indicate the fitting of the Farquhar et al. (1980) C3 model [23] with the straight line indicating the limitation to assimilation by RuBP carboxylation (*A_c_*) and the curved line indicating the limitation to assimilation by RuBP regeneration (*A_j_*) [24]. The data are for G/BMT control (**A**), 1-week (**B**), 2-week (**C**), or double (**D**) flood-treated plants, and for G/H2 control (**E**), 1-week (**F**), 2-week (**G**), or double (**H**) flood-treated plants. Bars indicate ± standard error of the means (*n* = 3).

In the control treatment (no flood), the *A/ci* data indicated that G/BMT had light- (1500 µmol photons m^−2^ s^−1^) and CO_2_-saturated (1600 µmol mol^−1^) rates of assimilation (*A_max_*) at 21.7 ± 1.7 µmol m^−2^ s^−1^, whereas at ambient CO_2_ (400 µmol mol^−1^), the rates of assimilation averaged 9.5 ± 1.4 µmol m^−2^ s^−1^ (Figure 4, Table 1). Consistent with this, the maximum rates of ribulose 1,5 bisphosphate (RuBP) carboxylation (*V_cmax_*) averaged 187 ± 17 µmol m^−2^ s^−1^ and the maximum rates of RuBP regeneration (*J_max_*) averaged 128 ± 13 µmol m^−2^ s^−1^ (Table 1). In G/BMT, 7 days of flooding (Treatment B, Figure 4B) caused a small, but insignificant, decrease in *A_max_* by 17% to an average of 17.1 ± 1.5 µmol m^−2^ s^−1^, whereas at 400 µmol mol^−1^ CO_2_, the rates of assimilation averaged 6.5 ± 1.5 µmol m^−2^ s^−1^, a 50% decrease in assimilation (*p* < 0.001). By contrast, this treatment had a minor (10%) effect on *V_cmax_*, averaging 169 ± 17 µmol m^−2^ s^−1^ and a smaller (3%) effect on *J_max_*, averaging 124 ± 13 µmol m^−2^ s^−1^.

Fourteen days of submersion (Treatment C, Figure 4C) had detrimental effects on G/BMT. *A_max_* was reduced by 75% to an average of 5.6 ± 1.4 µmol m^−2^ s^−1^ (*p* < 0.001) while the ambient rates of assimilation were negative (−0.95 ± 0.5 µmol m^−2^ s^−1^) (*p* < 0.001), indicating that respiration exceeded photosynthesis. Consistent with this, the maximum rates of RuBP carboxylation (*p* < 0.001) and regeneration (*p* < 0.001) were both severely reduced, in the range of 49 to 51 ± 8 µmol m^−2^ s^−1^, indicative of a 60–70% decrease (Figure 4C), in comparison to the control (see Figure 4A). The double flood treatment also resulted in negative rates of assimilation at ambient CO_2_, and a dramatic effect on *V_cmax_* and *J_max_*, which were barely responsive to internal CO_2_ (Figure 4D).

In comparison, G/H2 in the control treatment had ambient rates of assimilation within the same range as G/BMT (see Table 1). *A_max_* and *J_max_* were lower at 18.0 ± 1.1 µmol m^−2^ s^−1^ and 104 ± 6 µmol m^−2^ s^−1^, respectively, but this was not significant. However, *V_cmax_* was decreased by 34% (125 ± 13 µmol m^−2^ s^−1^) compared to G/BMT. Unlike G/BMT, 7 days of flooding (Treatment B, Figure 4F) had little effect on G/H2. At ambient CO_2_, assimilation rates averaged 6.3 ± 0.3 µmol m^−2^ s^−1^ and *A_max_* averaged 17.1 ± 0.5 µmol m^−2^ s^−1^, partially consistent with the control treatment. The maximum rates of RuBP carboxylation were slightly higher (14%) than the control and a similar result occurred for the RuBP regeneration (7%), but these were not significant. The 14-day flooding (Treatment C, Figure 4G) was not as detrimental on G/H2 as G/BMT, as the ambient CO_2_ rates averaged 6.3 ± 0.3 µmol m^−2^ s^−1^ and were largely consistent with the 7-day treatment. Similarly, the *A_max_* rates were also within the range of the control and 7-day treatment (Table 1), averaging 16.4 ± 1.3 µmol m^−2^ s^−1^. Furthermore, the maximum rates of RuBP carboxylation at 124 ± 12 µmol m^−2^ s^−1^ were comparable with the control treatment rates (Table 1) although the maximum rates of RuBP regeneration at 98 ± 4 µmol m^−2^ s^−1^ were slightly lower than the control rates, but not significantly. Despite the relatively little effects of the 7- and 14-day flood treatments, the double flood had similar extremely adverse consequences with negative assimilation rates, and *V_cmax_* and *J_max_* at less than 10% of control values (Figure 4H).

### 2.4. Effect of Photon Flux Density on Assimilation

**Figure 5:**
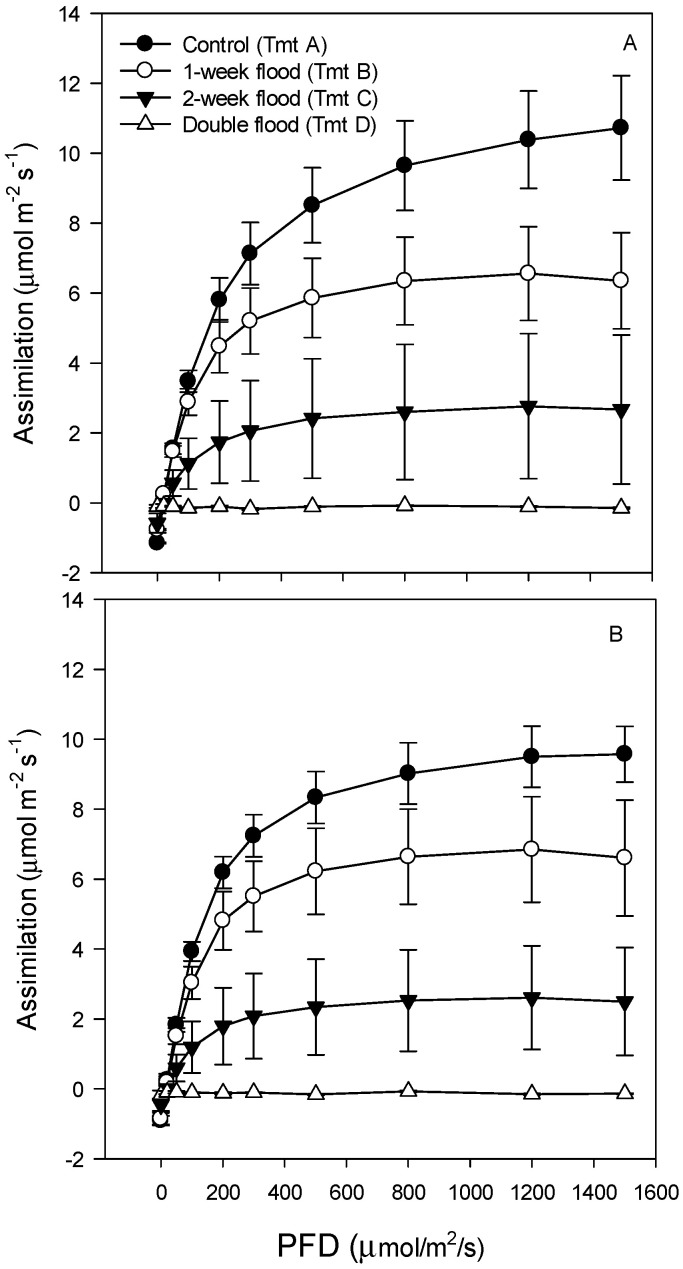
Assimilation in response to changes in the photon flux density (PFD) for G/BMT (**A**) and G/H2 (**B**) for each of the control and flood-treated plants. In each case, the lines are a fit to the hyperbolic tangent as described in Table 2, with an r^2^ = 0.98–0.99. Bars indicate ± standard error of the means (*n* = 4).

The light-saturated rates of assimilation (*P_max_*) at ambient CO_2_ (Figure 5, Table 2) for control G/BMT plants averaged 9.5 ± 0.4 µmol m^−2^ s^−1^ and fitted well to the light response curve [25] with an *r^2^* of 0.987. The photon yields averaged 0.0336 ± 0.0034 µmol mol^−1^ and dark respiration averaged 0.42 ± 0.33 µmol m^−2^ s^−1^. There was a certain effect of the 7 days of flooding on G/BMT with light-saturated assimilation (Figure 5A) decreasing by 33% to 6.64 ± 0.24 µmol m^−2^ s^−1^ compared to the control (*p* < 0.001). There was also a slight (7%) but non-significant decrease in the photon yield for this flood treatment while the dark respiration increased (10%) slightly but not significantly.

After the 14-day flood, the light-saturated assimilation averaged 2.67 ± 0.33 µmol m^−2^ s^−1^ (Figure 5A, Table 2), which was significantly lower than the control treatment and of the 7-day treatment (*p* < 0.001). The photon yield for the 14-day flooding treatment averaged 0.0272 ± 0.0036 µmol mol^−1^ and was lower than for the 7-day and control treatments, but not significantly. The rates of dark respiration were similar for all G/BMT treatments at about 0.4 µmol m^−2^ s^−1^. Leaves from the double flood treatment did not show a light response (Figure 5A), and therefore, these parameters could not be calculated.

The light-saturated assimilation for G/H2 averaged 9.36 ± 0.23 µmol m^−2^ s^−1^ (Table 2) and was not significantly different from G/BMT. The photon yield for G/H2 averaged 0.0363 ± 0.0042 µmol mol^−1^ and again was not significantly different from G/BMT. Similarly, the rates of dark respiration, although lower in the G/H2 than in G/BMT, were not significantly lower. Hence, in terms of the control, the light-dependent responses for assimilation were not inherently different between the two rootstocks. Like G/BMT, the 7-day flooding treatment (Figure 5B) caused a significant decline in light-saturated assimilation (7.1 ± 0.24 µmol m^−2^ s^−1^) for G/H2 compared with the control. There were again no significant differences in the photon yield or dark respiration between the two treatments. When G/H2 was exposed to 14 days of root zone flooding, there was a marked and significant decrease in light-saturated assimilation (Figure 5B, Table 2), averaging 2.3 ± 0.22 µmol m^−2^ s^−1^, not just significantly different with the control but also with the 7-day treatment (*p* < 0.001). Like G/BMT, leaves of the double flood treatment did not show a light response and assimilation rates remained negative (Figure 5B).

### 2.5. Cambium Vitality

Cambium vitality above and below the graft was not affected by the 1-week flood treatment in either G/BMT or G/H2 with 100% of the plants remaining vital (*n* = 5). Likewise, the two-week flood resulted in no immediate loss in cambium vitality in either the scion or rootstock. However, when assessed 1 month later, 80% of G/BMT scions had lost cambium vitality, while only 20% of G/H2 scions had lost cambium vitality. The double flood treatment resulted in 40% of the plants losing scion vitality while submerged, and this increased to 80% of plants, regardless of rootstock, one month later. The cambium below the graft remained vital in all plants during the double flood, but one month later, 20% of G/BMT plants had lost vitality. In contrast, G/H2 plants did not show any loss in cambium vitality below the graft one month later.

### 2.6. Total Non-Structural Carbohydrates

**Figure 6:**
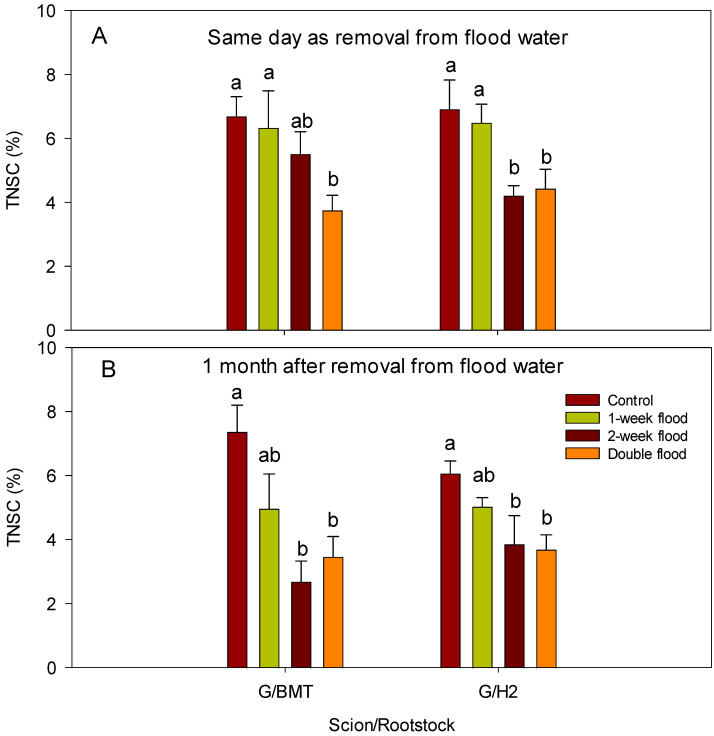
Trunk stored total non-structural carbohydrates (TNSC) of G/BMT and G/H2 in response to a 1-week flood, 2-week flood, or double flood treatment. Measurements were conducted when plants were removed from the water (**A**), and 1 month later (**B**). Bars indicate ± standard error of the means (*n* = 5). Flood treatment was significant (*p* < 0.001), but rootstock was not (*p* = 0.503) at either assessment time. There was also no significant interaction of treatment x rootstock (*p* = 0.527). Groups that share a letter are not significantly different, while groups with completely different letters are significantly different.

Trunk total non-structural carbohydrates (TNSC) were affected by prolonged full submersion (Figure 6). The 1-week flood treatment did not result in a significant decline in trunk TNSC (% of dry weight) in either rootstock immediately upon the removal from the flood water, as well as 1 month later. The 2-week flood resulted in a decline from 6.9% to 4.2% TNSC in G/H2 (*p* < 0.001) but it did not decline further over the following month. In contrast, G/BMT did not have an immediate decline in TNSC after 2 weeks of flooding, but 1 month later it had lost 63% of its starch reserves relative to the control, with a decline to 2.7% of plant dry weight (*p* < 0.001). Immediately following the double flood, both rootstocks had undergone a substantial decline in TNSC to 4.4% and 3.7% for G/H2 and G/BMT, respectively (*p* < 0.001; Figure 6). Over the subsequent month, the TNSC did not decline any further.

### 2.7. Canopy Mortality

**Figure 7:**
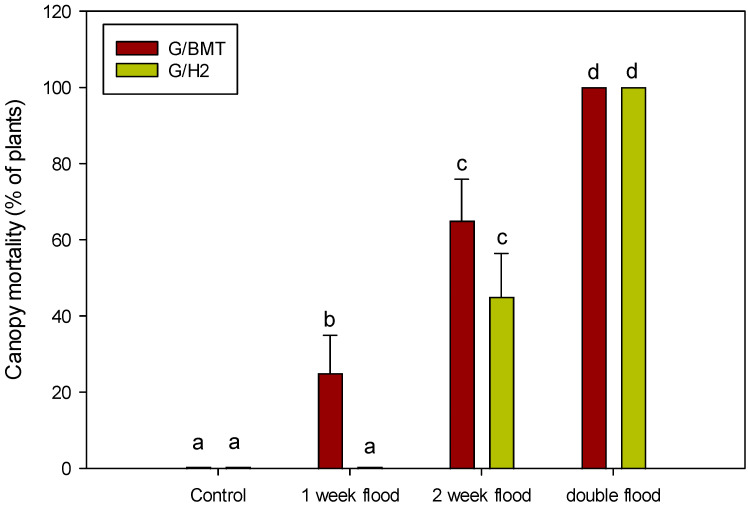
Canopy mortality of G/BMT and G/H2 in response to no flooding (control), a 1-week flood, 2-week flood, or a double flood treatment, as assessed 3 months after the last submersion. None of the control plants underwent canopy death. Bars indicate ± standard error of the means (*n* = 5). Flood duration (*p* < 0.001) was significant, as was rootstock (*p* = 0.013). There was no significant interaction of rootstock by flood duration *(p* = 0.095). Groups that share a letter are not significantly different, while groups with completely different letters are significantly different.

Flood duration was highly significant (*p* < 0.001) to canopy mortality (Figure 7). Rootstock also had a significant effect (*p* < 0.013). When assessed 3 months following submersion, H2 had better scion (leaves and branches) survival rates with no canopy death in the 1-week flood treatment relative to 25% in BMT. The 2-week flood treatment did not result in differences in canopy mortality. Similarly, scion death was 100% after the double flood in both rootstocks.

The photosynthetic parameters *A*_400_, *A_max_*, *V_cmax_*, and *J_max_* were highly correlated with canopy survival (*R*^2^ = 0.86, 0.90, 0.85, and 0.89, respectively, *p* < 0.001), as was *g_s_* (*R*^2^ = 0.71, *p* < 0.01). Canopy survival was also highly correlated with trunk TNSC upon removal from the flood water (*R*^2^ = 0.78, *p* < 0.01), and one month later (*R*^2^ = 0.63, *p* < 0.05). As expected, plant total leaf dry weight was highly correlated with plant survival one month after removal from the water (*R*^2^ = 0.86, *p* < 0.001). However, there was no relationship between canopy survival and root dry weight at the time the plants were removed from the water (*R*^2^ = 0.29, *p* = 0.165) or one month later (*R*^2^ = 0.21, *p* = 0.249).

## 3. Discussion

This study assessed the effect of flooding on young macadamia plants. The plants were able to tolerate 1 week of full submersion with no symptoms of severe stress after removal from the water and exposure to sprinkler irrigation. However, the 2-week and double flood treatments were detrimental to canopy retention and photosynthetic capacity of the remaining leaves. Leaf abscission is a survival mechanism to reduce the metabolic cost of maintaining the unproductive component of the canopy [26], and it may also allow the reallocation of resources to new growth, stems and roots [27]. The plant may thus improve survival of the remaining tissues and the capacity for regrowth once favourable conditions return. However, canopies continued to decline over 3 months after removal from the prolonged flood treatment, despite favourable conditions, with the double flood treatment eventually triggering the death of the entire scion, regardless of rootstock. The ability to recover from hypoxic conditions may be the result of changes in metabolic processes or carbohydrate allocation to avoid energy collapse [28]. Additionally, ethylene sensitivity, ABA levels, and cytokinin transport [29,30,31] can determine the speed and nature of the flooding response. Finally, the capacity to recover may also stem from differences in water regulation. Oxygen deprivation can inhibit aquaporin activity [32], resulting in lower leaf water potential and reduced stomatal conductance [33]. Furthermore, hydraulic and chemical signalling may induce stomatal closure when root hydraulic activity declines [34]. Varying sensitivity to hypoxia was evident in *Prunus* rootstocks where flooding led to differences in *g_s_* [35]. In our study, however, *g_s_* was strongly downregulated in both rootstocks, indicating that stomatal behaviour does not account for the differences observed between them.

One aim of the study was to examine how whole-plant submersion affected leaf-level carbon assimilation once the flood had receded. We specifically examined the responses of photosynthetic assimilation (*A*), maximum rates of ribulose 1,5 bisphosphate (RuBP) carboxylation (*V_cmax_*), and RuBP regeneration (*J_max_*) under both ambient and saturating CO_2_ conditions, as well as response to photon flux density (PFD). The findings provide insights into how flooding can impair photosynthetic capacity and how a rootstock may impart an effect on the scion after flooding stress.

The results demonstrated that G on both rootstocks showed a significant decline in assimilation rates (*A_max_*) following whole-plant submersion, with the response being more pronounced in G/BMT than in G/H2. Under control conditions, G/BMT exhibited a high rate of photosynthesis at both saturating CO_2_ (*A_max_* = 21.7 µmol m^−2^ s^−1^) and ambient CO_2_ (*A*_400_ = 9.5 µmol m^−2^ s^−1^), consistent with typical C3 plant responses to light and CO_2_ availability. However, after 7 days of flooding, there was a major reduction in the stomatal conductance (between 45 and 68%), with G/BMT expressing greater stomatal closure. Consistent with a reduced CO_2_ gradient, G/BMT revealed a 17% decrease in *A_max_* and a marked 50% decrease in *A*_400_, suggesting that even short-term flooding induced significant metabolic stress that limited the plant’s ability to assimilate carbon. This reduction in assimilation was also accompanied by a minor decrease in the maximum rates of RuBP carboxylation (*V_cmax_*) and RuBP regeneration (*J_max_*), indicating that while photosynthetic metabolism was affected, the overall capacity for carboxylation and regeneration remained relatively intact under shorter flooding durations. In contrast, G/H2 exhibited the smaller reduction in stomatal conductance with a consequence of a smaller decrease in assimilation, following the 7-day flood treatment, with *A_max_* remaining almost unchanged compared to the control (17.1 µmol m^−2^ s^−1^). G on this rootstock also showed a slight increase in *V_cmax_* and *J_max_*, suggesting that it may have a greater tolerance to flooding-induced stress, at least in the short term.

Dramatic reductions in photosynthetic capacity occurred after 14 days of flooding and the double flood treatment. After 14 days of flooding, the stomatal conductance had decreased for G on both rootstocks by 79–82%, indicating a restrictive pathway for CO_2_ to enter leaf metabolism. Consistent with this, for G/BMT, *A_max_* decreased by 75%, and assimilation at ambient CO_2_ (*A*_400_) became nearly negative, indicating a near-total shutdown of photosynthetic metabolism. Furthermore, there was a corresponding severe reduction in *V_cmax_* and *J_max_*, both of which decreased by approximately 60–70%. The almost complete loss of stomatal conductance and parallel impaired photosynthetic function in G/BMT after 14 days of flooding suggests that this rootstock was highly susceptible to extended submersion and anaerobic conditions. RuBP carboxylation (*V_cmax_*), which was affected by reduced CO_2_ availability, was also partially controlled by nitrogen (N) [36], and reduced root function may lead to impaired N uptake. Furthermore, antioxidant capacity and the accumulation of ROS [37], reduced ATP availability [38], and damage to the thylakoid membranes [6] that are associated with flooding may have affected RuBP regeneration and electron transport rate.

In contrast, both light-saturated and ambient CO_2_ G/H2 assimilation remained relatively stable after 14 days of flooding, although it did have a moderate decline, despite the marked decrease in stomatal function. The maximum rates of RuBP carboxylation and regeneration were somewhat lower but did not show the same dramatic reduction apparent in G/BMT. These results suggest that G/H2 may have inherent physiological metabolism that confers greater tolerance to flooding, perhaps such as more efficient oxygen transport or enhanced root adaptation to waterlogged conditions. Additional measurements such as root porosity, aerenchyma formation, root respiration, ROS markers, or hormone profiles are required to support this hypothesis. Of relevance, given the parallels in stress responses between drought and flood [39], H2 was identified to have high drought tolerance in a study of 14 cultivars that assessed 13 physiological indices, including those of ROS metabolism [40]. Nevertheless, even G/H2 was not immune to the effects of prolonged flooding in our study, as indicated by the significant decrease in stomatal function and in light-saturated assimilation after 14 days. There was also a marked decline in both *V_cmax_* and *J_max_*, although not to the same extent as G/BMT. These differences highlight the variability in flooding tolerance between the two rootstocks, with G/H2 showing more resilience in the early stages of stress but still suffering substantial reductions in photosynthetic capacity after prolonged flooding. That said, stomatal function and photosynthetic capacity was completely lost after the double flood in both rootstocks. The dramatic loss in leaf function likely resulted in canopy mortality as *A*_400_, *A_max_*, *V_cmax_*, and *J_max_* were all highly positively correlated with plant survival. A decrease in photosynthesis and chlorophyll fluorescence (*Fv/Fm*) was apparent in *Typha domingensis* (southern cattail) with increasing flooding depth [41]. Like our results, when plants were exposed to deepwater flooding, leaves did not recover and plants eventually died.

The light-saturated rates of assimilation (*P_max_*) and photon yield showed a clear response to both short- and long-term flooding, though this response varied between rootstocks. For G/BMT, the 7-day flood treatment reduced light-saturated assimilation by 33%, with a slight increase in dark respiration. This increase in dark respiration suggests an attempt by the plant to maintain some energy balance post-flooding, although the overall effect on assimilation was negative. Prolonged flooding (14 days) had a less pronounced effect on photon yield, hence maintained light interception, but caused a minor additional reduction in assimilation, highlighting that long-term stress reduced the efficiency of the plant’s photosynthetic machinery. Similarly, for G/H2, the 7-day flooding treatment caused a significant decrease in light-saturated assimilation, and the rates of dark respiration were significantly higher compared to the control. Interestingly, the 14-day flooding treatment also resulted in a minor drop in assimilation, with photon yield remaining relatively low, pointing to a further decline in photosynthetic efficiency and light interception. These changes in photon yield might suggest leaf surfaces were damaged, impairing stomata and light interception. Coupled with the increases in dark respiration, this suggests a shift in the plant’s metabolic processes, possibly reflecting energy demand from existing carbohydrate reserves in response to the stress. Both double-flooded rootstocks showed no response to PFD, suggesting complete loss in light interception and hence in photosynthetic capacity. It is evident that new leaves are required for whole-plant carbon assimilation to return to original levels.

The consumption of plant TNSC during submersion suggests that the plants did not adopt a quiescence or dormancy strategy and indicates that there was enough oxygen present within the tissues to stimulate its breakdown to soluble sugars. The two rootstocks had similar levels before and after the various periods of submersion. Although studies report that substantial starch and total soluble carbohydrate reserves before submergence significantly increase survival rates [42,43], the consumption rate during submergence may also be important [44]. In our study, there was a strong correlation between canopy survival and TNSC concentrations upon plant removal from the floodwater. Continued TNSC degradation after re-exposure to oxygen suggests that it was an energy source for repair and recovery, and possibly cell division for new tissue regeneration, since C assimilation was depressed. In support of this, new shoots emerged below the graft union in plants where the scion was no longer alive. Conversely, the reduction in root biomass during and after submersion indicates that new root growth did not keep pace with the breakdown and loss of older, damaged roots. This suggests that there was insufficient energy to mobilise the remaining carbohydrates to the below-ground tissues. Interestingly, the double flood treatment did not generate a substantial decline in trunk TNSC over the month following emergence, despite the absence of any photosynthetic canopy. This is likely due to the overall loss of scion function, which eliminated the energy demands that would normally be associated with maintaining an active canopy.

This study provides valuable insights into the physiological consequences of flooding on macadamia carbon metabolism, photosynthetic performance, and TNSC dynamics. Grafted plants of G on both BMT and H2 rootstocks exhibited a pronounced sensitivity to flooding, with dramatic reductions in canopy volume, carbon assimilation, *V_cmax_*, and *J_max_* after extended and repeated exposure. Progressive canopy loss following flooding likely reflects an inability to sustain leaf function once carbohydrate reserves are depleted, resulting in reduced carbon supply to support maintenance respiration and post-flood recovery. Rootstock-mediated variation in carbohydrate storage capacity, mobilisation efficiency, and post-hypoxic recovery of root function may directly influence the ability of the scion to retain a canopy and sustain photosynthesis following inundation.

## 4. Materials and Methods

### 4.1. Plant Submergence

**Figure 8:**
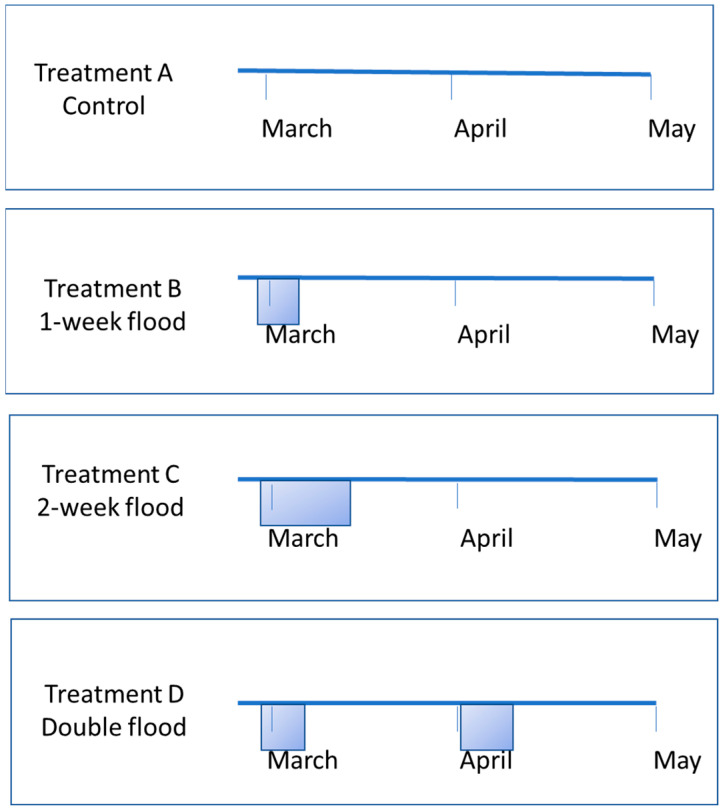
Timeline of full plant submersion. Control plants remained untreated (Treatment A). Plants were flooded for 1 week (Treatment B) or 2 weeks (Treatment C). Treatment D consisted of 2 floods, one month apart, with the first flood lasting 1 week and the second lasting 10 days.

One year-old *Macadamia integrifolia* plants of the cultivars MIV1-G, MCT1, 344, A203, and 849, grafted on pure H2 (*M. integrifolia*) and the hybrid Beaumont (BMT) (*M. integrifolia* x *M. tetraphylla)* rootstock, were purchased from a local nursery (*n* = 120 plants for each scion/rootstock combination with 1200 plants in total). They were re-planted in 6 L pots containing alluvial floodplain soil consisting of a mixture of silt, sand and clay and left to settle under sprinkler irrigation (3 times daily, 20 min). After 2 months, the plants were subjected to one of four treatments (*n* = 30). One set of plants remained outdoors under sprinkler irrigation (Control, Treatment A). The rest of the plants were submerged (including the entire canopy) for 7 days (Treatment B), 14 days (Treatment C), or exposed to a double flood consisting of 7 days of flooding, followed by 1 month of no flooding, then followed by another 10 days of flooding (Treatment D; Figure 8). Treatment D simulated the 2022 flood that occurred in the Northern Rivers of NSW. The plants were randomly allocated to 1 of 10 skip bins (6 m^3^, 3.60 m long, 1.50 m wide and 1.25 m deep) in a randomised block design with each skip bin containing 3 plants of each treatment and each scion/rootstock combination, and a total of 90 plants per bin. Once the plants were removed from the bins, they were placed under sprinkler irrigation with the plants of treatment A in natural daylight and temperature conditions. Due to logistical time constraints, only plants of G/BMT or G/H2 were used for detailed photosynthesis measurements. Similarly, due to resource constraints, plant carbohydrate assessments were limited to this combination. MIV1-G is a relatively new variety that is recommended for the region where the study was conducted. It is a representative, stable cultivar with medium vigour, moderate canopy density, and medium to good kernel recovery. It is an *M. integrifolia* species, the dominant species in commercial production and it has good compatibility with several rootstocks. H2 and BMT were used as the rootstocks as these are commonly used where flooding occurs.

### 4.2. Environmental Conditions

**Figure A1:**
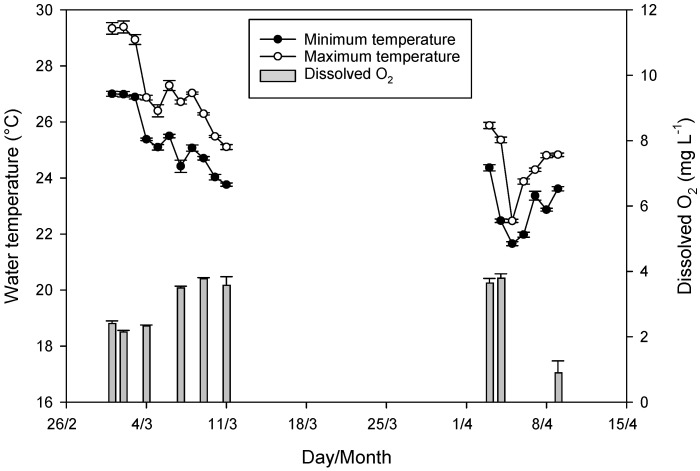
Water temperature and dissolved O_2_ levels during the flood treatments. The first flood occurred in early March and the second flood occurred in early April.

The bins were filled with dam water. Water quality was monitored in each bin 2 to 3 times per week at mid-canopy height with a portable Horiba water quality meter (U-51, Instrument Choice, Adelaide, Australia). Water pH averaged 8.17 ± 0.06, dissolved O_2_ 3.0 ± 0.3 mg/L, and total dissolved solids 66.6 ± 2.1 mg L^−1^. A Tinytag submersible temperature sensor (TG-4100, Hastings Data Loggers, Port Macquarie, Australia) was placed at mid-plant height in 3 bins and logged every half hour. The average minimum water temperature was 23.6 ± 0.3 °C and the average maximum water temperature was 27.9 ± 0.6 °C (Figure A1). Light was able to penetrate the water and reach the upper part of the canopy.

### 4.3. Gas Exchange

Between 7 and 14 days post-flooding, the photosynthetic response to intercellular CO_2_ (*A/ci*) was measured with the LI6400 XT gas exchange system (LiCor Biosciences, Lincoln, Nebraska).

On all occasions, fully expanded leaves were measured. Measurements were conducted at constant leaf temperatures and averaged between 25 and 27 °C throughout all measurements. During each *A/ci* response, the photon flux density (PFD) was maintained at 1500 µmol m^−2^ s^−1^, and the reference CO_2_ was initially set at 400 µmol mol^−1^ to ensure photosynthetic rates were steady. The CO_2_ concentration was then reduced in 50–100 µmol mol^−1^ steps to 50 µmol mol^−1^ then increased back to 400 µmol mol^−1^ to ensure the photosynthetic rates had increased back to the original rate. Subsequently, the reference CO_2_ was increased in 100–200 µmol mol^−1^ steps to a maximum CO_2_ concentration of 1600 µmol mol^−1^. The vapour pressure deficits (leaf to air) were not controlled but remained between 1.6 and 1.9 kPa during the measurements. For G on each rootstock, the *A/ci* procedure was repeated 3 times and a new leaf was used for each procedure.

Over the same duration, the photosynthetic response to PFD was also measured with the LI6400 XT. On each occasion, the PFD was initially set at 1500 µmol (photons) m^−2^ s^−1^ and when photosynthetic rates were steady, the PFD was reduced in 100–200 µmol (photons) m^−2^ s^−1^ steps to approximately 1 µmol (photons) m^−2^ s^−1^ (darkness). During each measurement, the leaf temperature ranged between 25 and 29 °C and the reference CO_2_ concentration was maintained at 400 µmol mol^−1^. The vapour pressure deficits were not controlled but remained between 1.5 and 2.0 kPa. The whole measurement procedure was repeated three times for G on both rootstocks, with a new fully expanded leaf for each light response on each occasion.

### 4.4. Cambium Vitality

Small shallow windows were cut into the bark of the trunk above the graft and at three points below the graft, in the upper, mid and lower portions of the trunk. Once the cambial zone was revealed, a visual assessment was carried out with a healthy cambium consisting of moist tissue of a light green colour. A cambium was categorised as compromised if it was dry and had the absence of green colour. Assessments were carried out for each treatment immediately after the plants were removed from the water and 30 days later (*n* = 5).

### 4.5. Total Non-Structural Carbohydrates

Plants (*n* = 5) from G/BMT and G/H2 were destructively harvested after the removal from the flood waters, and again 30 days later. The entire trunk below the graft union was dried at 60 °C and then ground to a fine powder (<0.8 mm) before TNSC analysis at a commercial laboratory (AgEnviro Labs, Wagga Wagga, Australia). TNSC was determined by a two-step enzymatic digestion, without pre-extraction of soluble sugar fractions. Starches were quantitatively converted to glucose through the sequential action of alpha-amylase and amyloglucosidase. The filtered extracts of hydrolysed starches and endogenous sugars were then analysed by flow injection analysis using the alkaline ferricyanide decolouration method. This method involved the treatment of the extract with heat (90 °C) and 1 M hydrochloric acid to yield invert sugars, which were then dialysed into a stream of alkaline potassium ferricyanide. At that point, the sugars reduced the yellow ferricyanide to colourless ferrocyanide and the measured absorbance at 420 nm was directly proportional to the invert sugar content, expressed as glucose equivalent.

### 4.6. Data Analysis

Plant dry weight, TNSC and gas exchange data were analysed using factorial analysis of variance (ANOVA) in Genstat (v. 23.1; VSN International, Hemel Hempstead, UK). The experimental design consisted of two rootstocks and four flood treatments (control, 1-week flood, 2-week flood, and double flood). To account for potential non-independence arising from shared flooding exposure within bins, bin was included as a random blocking factor in all mixed-model analyses. Individual plants were considered subsamples within bins for treatment effects rather than fully independent experimental units. Only G/BMT and G/H2 were used in the analysis to align with the photosynthesis data. Canopy mortality count data were analysed using a generalised linear mixed model (GLMM). The model was fitted assuming a Poisson distribution with a log link function. Means were separated using Fisher’s protected least significant difference (LSD) test at *p* ≤ 0.05, where treatment effects were significant.

For fitting the *A/ci* data to the C_3_ photosynthesis model of Farquhar et al. [23], the apparent maximum rates of RuBP carboxylation (*V_cmax_*) and the maximum rates of RuBP regeneration (*J_max_*) were determined using the *A/ci* curve fitting facility of Sharkey et al. [24]. As there were no independent methods of determining the mesophyll conductance during these measurements, all data were estimated using the intercellular CO_2_ concentration.

The light responses were each analysed using non-linear regression using Origin Pro 8 (OriginLab Corporation, Northampton, MA, USA) to fit a hyperbolic tangent function according to Greer and Halligan [25].

## 5. Conclusions

Collectively, these findings highlight the importance of carbohydrate availability as a central determinant of flood tolerance in macadamia. They further indicate that identifying rootstocks with enhanced capacity for carbohydrate storage during favourable conditions, as well as efficient reserve mobilisation during stressful periods, may be key to improving tree survival and productivity in a flood-prone environment. Future research should therefore expand to a broader range of rootstock–scion combinations and focus on the mechanistic links between carbohydrate dynamics and canopy recovery following flooding events.

## Figures and Tables

**Table 1:** Mean and standard errors (*n* = 3) of the assimilation at limiting CO_2_ (*A*_400_) and at saturating CO_2_ (*A_max_*) and the maximum rate of RuBP carboxylation (*V_cmax_*) and the maximum rate of RuBP regeneration (*J_max_*) for cv. G on two rootstocks in the control and the flood treatments. Flood treatment was significant for *A*_400_ (*p* < 0.001), *A_max_* (*p* < 0.001), *V_cmax_* (*p* < 0.001), and *J_max_* (*p* < 0.001). The main effect of rootstock was not significant for any parameter. The rootstock by treatment interaction was significant for *A*_400_ (*p* < 0.05), *A_max_* (*p* < 0.05), *V_cmax_* (*p* < 0.05), and *J_max_* (*p* < 0.05). Means that share a letter are not significantly different, while means with completely different letters are significantly different.

Cultivar/Rootstock	Treatment	A_400_	A_max_	V_cmax_	J_max_
G/BMT	A-Control	9.45 ± 1.37 a	21.69 ± 1.66 a	187 ± 17 a	128 ± 13 a
	B-1-week flood	6.48 ± 1.45 b	17.14 ± 1.45 a	169 ± 15 ab	124 ± 13 a
	C-2-week flood	−0.95 ± 0.52 c	5.55 ± 1.42 b	51.5 ± 17 d	49 ± 12 b
	D-Double flood	−1.21 ± 0.11 c	0.95 ± 0.47 c	4.25 ± 0.28 e	8.94 ± 0.41 c
G/H2	A-Control	9.11 ± 0.66 a	18.0 ± 1.08 a	125 ± 13 cb	104 ± 11 a
	B-1-week flood	6.28 ± 0.32 b	17.13 ± 1.1 a	144 ± 12 abc	112 ± 5 a
	C-2-week flood	6.27 ± 1.24 b	16.4 ± 0.95 a	124 ± 13 cb	98 ± 4 a
	D-Double flood	−1.11 ± 0.24 c	1.60 ± 0.62 bc	5.05 ± 3.09 e	9.34 ± 0.57 c

**Table 2:** Assimilation responses to changes in photon flux density (PFD) for G on two rootstocks, exposed to flooding treatments. Photosynthesis at saturating CO_2_ (*P_max_*) is estimated from the non-linear analysis of the hyperbolic tangent fitted to these data, as is the photon yield and the dark respiration. The data are means ± SE (*n* = 4 except for the 14-day flood treatment where *n* = 2–3). Parameters could not be estimated for Treatment D as there was no response to PFD. Flood treatment was significant for *P_max_* (*p* < 0.001) only. The main effect of rootstock and the interactive effect of rootstock by treatment was not significant for any parameter. Means that share a letter are not significantly different, while means with completely different letters are significantly different.

Cultivar/Rootstock	Treatment	P_max_	Photon Yield	Dark Rs
G/BMT	A-Control	9.47 ± 0.55 a	0.034 ± 0.003 a	0.42 ± 0.33 a
	B-1-week flood	6.64 ± 0.24 b	0.031 ± 0.003 a	0.46 ± 0.21 a
	C-2-week flood	2.67 ± 0.33 c	0.027 ± 0.004 a	0.40 ± 0.29 a
G/H2	A-Control	9.36 ± 0.04 a	0.036 ± 0.004 a	0.33 ± 0.09 a
	B-1-week flood	7.09 ± 0.24 b	0.037 ± 0.003 a	0.56 ± 0.21 a
	C-2-week flood	2.32 ± 0.22 c	0.029 ± 0.003 a	0.51 ± 0.11 a

## Data Availability

The original contributions presented in this study are included in the article. Further inquiries can be directed to the corresponding author.

## Abbreviations

The following abbreviations are used in this manuscript:

*A*	photosynthetic rate
*A*_400_	photosynthesis at ambient CO_2_
*A_max_*	photosynthesis at saturating CO_2_
*A/ci*	photosynthetic response to intercellular CO_2_
*A_c_*	the limitation to assimilation by RuBP carboxylation
*A_j_*	the limitation to assimilation by RuBP regeneration
*g_s_*	stomatal conductance
*J_max_*	Ribulose 1,5 bisphosphate (RuBP) regeneration
PFD	photon flux density
*P_max_*	light saturated rates of assimilation
RuBP	Ribulose 1,5 bisphosphate
TNSC	total non-structural carbohydrates
*V_cmax_*	Ribulose 1,5 bisphosphate (RuBP) carboxylation
